# Maternal OGTT Glucose Levels at 26–30 Gestational Weeks with Offspring Growth and Development in Early Infancy

**DOI:** 10.1155/2014/516980

**Published:** 2014-02-13

**Authors:** Gongshu Liu, Nan Li, Shurong Sun, Jing Wen, Fengjun Lyu, Wen Gao, Lili Li, Fang Chen, Andrea A. Baccarelli, Lifang Hou, Gang Hu

**Affiliations:** ^1^Tianjin Women's and Children's Health Center, Tianjin 300070, China; ^2^Chronic Disease Epidemiology Laboratory, Pennington Biomedical Research Center, 6400 Perkins Rd, Baton Rouge, LA 70808, USA; ^3^Departments of Epidemiology and Environmental Health, Harvard School of Public Health, Boston, MA 02115, USA; ^4^Department of Preventive Medicine, Feinberg School of Medicine, Northwestern University, Chicago, IL 60611, USA

## Abstract

*Aims*. We aim to evaluate the association of maternal gestational oral glucose tolerance test (OGTT) glucose concentrations with anthropometry in the offspring from birth to 12 months in Tianjin, China. *Methods*. A total of 27,157 pregnant women underwent OGTT during 26–30 weeks gestation, and their children had body weight/length measured from birth to 12 months old. *Results*. Maternal OGTT glucose concentrations at 26–30 gestational weeks were positively associated with Z-scores for birth length-for-gestational age and birth weight-for-length. Compared with infants born to mothers with normal glucose tolerance, infants born to mothers with gestational diabetes mellitus (impaired glucose tolerance/new diabetes) had higher mean values of Z-scores for birth length-for-gestational age (0.07/0.23; normal group −0.08) and birth weight-for-length (0.27/0.57; normal group −0.001), smaller changes in mean values of Z-scores for length-for-age (0.75/0.62; normal group 0.94) and weight-for-length (0.18/−0.17; normal group 0.37) from birth to month 3, and bigger changes in mean values in Z-scores for weight-for-length (0.07/0.12; normal group 0.02) from month 9 to 12. *Conclusions*. Abnormal maternal glucose tolerance during pregnancy was associated with higher birth weight and birth length, less weight and length gain in the first 3 months of life, and more weight gain in the months 9–12 of life.

## 1. Introduction

Gestational diabetes mellitus (GDM) is increasingly common worldwide [1]. In China, the prevalence of GDM has increased from 2.4% in 1999 to 6.8% in 2008 [2], now close to the US level. One of the major concerns about GDM is that it may be contributing to a vicious intergenerational cycle of obesity and diabetes [3]. Women with a history of GDM are at increased risk of type 2 diabetes and impaired glucose tolerance (IGT) later in life [4], especially at the first 5 years after delivery [5]. The exposure to diabetes during pregnancy is associated with increased risks of neonatal adiposity, childhood obesity, insulin resistance, IGT, and type 2 diabetes in the offspring in some but not all studies [6–8].

Childhood obesity is a global problem. The prevalence of childhood obesity is 6.7% in 2010 worldwide, and 70% of obese adolescents become obese adults [9]. Hillier et al. [10] found that a higher hyperglycemia level in pregnancy was associated with an increased future risk of obesity in their children at 5–7 years. The Hyperglycemia and Adverse Pregnancy Outcome (HAPO) study found a weak association between maternal glucose during pregnancy and obesity in the offspring at age 2 [11]. However, most previous studies have paid more attention to the association between maternal hyperglycemia and children obesity in the offspring of more than 5 years old [8]. Few studies have examined whether abnormal maternal glucose tolerance during pregnancy also predicts weight gain in early infancy [12]. It has been suggested that rapid weight gain in infancy (<2 years old) predicts a later risk of obesity in childhood and adulthood [13]. The aim of the present study was to evaluate the association of maternal OGTT glucose levels at 26–30 gestational weeks with anthropometry in the offspring from birth to 12 months in Tianjin, China.

## 2. Methods

### 2.1. Study Sample

Tianjin is the fourth largest city with over 12.9 million residents in Northern China, and 4.3 million residents live in central urban districts. The prenatal care and children health care in central urban districts are a routine of a three-tier care system consisting of approximately 65 primary hospitals, 6 district-level Women's and Children's Health Centers, and a city-level (Tianjin) Women's and Children's Health Center (also including tertiary hospitals). In Tianjin, all pregnant women are registered at the primary hospitals, and in the 32nd gestational week, they are referred to a secondary hospital or a tertiary hospital for management till delivery. All children are given the health examinations in the newborns (<3 days after birth), postnatal period (42 days after birth), infancy (<3 years old), and preschool (3–7 years old). Tianjin Women and Children's Health Centre is the leader of the 3-tier care system and responsible for organization, coordination, and implementation of women and child health care, research, and promotion projects.

Health care records for both pregnant women and their children have been collected and available in electronic form since 2009 [14]. Pregnant women health records start within the first 12 weeks of pregnancy and include general information (age, occupation, education, smoking habits, etc.), history of diseases, family history of diseases, clinical measurements (height, weight, blood pressure, gynaecological examinations, ultrasonography, GDM screening test, and other lab tests), complications during pregnancy, pregnancy outcomes (delivery modes, labor complications, etc.), and postnatal period examinations (<42 days after delivery, etc.). Children health records include information from newborns (date of birth, sex, gestational week of birth, birth weight, birth length, etc.), postnatal period, and infancy. The information of feeding modalities during the first 6 months and the measurements of recumbent length/height, weight are collected and available in each health examination. Between July 2009 and June 2011, 43,854 mother-child pairs' health care records were available in central urban districts. The present study included 27,157 mothers (61.9%) with all information, who underwent oral glucose tolerance test (OGTT) during 26–30 weeks of gestation after excluding 33 mothers who were diagnosed with diabetes before pregnancy, 3,734 mothers missing glucose challenge test (GCT), 2,689 mothers missing OGTT, and 10,241 mother-child pairs missing any variables required for this analysis. Compared with children excluded in the present study, the children included in the present analysis had similar age (12.2 versus 12.2 months old), there were fewer males (51.9% versus 52.8%), and their mothers were older (28.0 versus 27.0 years old). Of 27,157 mothers, the rates of child health examination at months 3, 6, 9, and 12 were 88.4%, 90.7%, 94.5%, and 98.3%, respectively. The study and analysis plan were approved by Tianjin Women's and Health's Health Center Institutional Review Boards.

### 2.2. Glucose Testing and GDM Diagnosis

A universal screening for GDM has become an integral part of the antenatal care in urban Tianjin [15]. A total of 27,157 pregnant women at 26–30 gestational weeks underwent OGTT. If pregnant women had a 1-hour 50 g GCT level ≥7.8 mmol/L, they were asked to undergo a 75 g 2-hour OGTT for GDM diagnosis test at Tianjin Women's and Children's Health Center. OGTT results were interpreted according to World Health Organization (WHO) diagnostic criteria [16]. Pregnant mothers were regrouped into four categories based on the results of the glycemic screening tests: (1) normal glucose tolerance, defined as normal results of the GCT (<7.8 mmol/L); (2) failed GCT (≥7.8 mmol/L) with normal results on the OGTT, fasting glucose <6.1 mmol/L and 2-hour glucose <7.8 mmol/L; (3) impaired glucose tolerance (IGT), defined as failed GCT and fasting glucose <7.0 mmol/L and 2-hour glucose ≥7.8 and <11.1 mmol/L; and (4) newly diagnosed diabetes (new DM), defined as failed GCT and fasting glucose ≥7.0 mmol/L or 2-hour glucose ≥11.1 mmol/L. In the present study, 14 pregnant mothers were defined as isolated impaired fasting glucose (IFG), failed GCT and fasting glucose ≥6.1 and <7.0 mmol/L and 2-hour glucose <7.8 mmol/L, and these 14 mothers were included in the IGT group and treated as IGT. GDM was defined as women with IGT (*n* = 1262)/IFG (*n* = 14) or new DM (*n* = 144) during 2 h OGTT.

### 2.3. Measurements

Mothers' anthropometric data were collected during the pregnancy by obstetricians in the primary hospitals. Weight and height were measured in light clothing and no shoes using a beam balance scale (RGZ-120, Jiangsu Suhong Medical Instruments Co., China). Blood pressure was measured using a standardized mercury sphygmomanometer (XJ11D, Shanghai Medical Instruments Co., China). Children's weight and length were measured at birth, 3 months (<4 months), 6 months (≥4 and <7 months), 9 months (≥7 and <10 months), and 12 months (≥10 and <13 months). Weight was measured to the nearest 0.01 kg using a digital scale (TCS-60, Tianjin Weighing Apparatus Co., China). Length was measured to the nearest 0.1 cm using a recumbent length stadiometer (YSC-2, Beijing Guowangxingda, China). We have done a validity study to compare the electronic data of measurements of birth weight and hospitals' measurements of birth weight among 454 children in six major hospitals. The correlation between two measurements is 0.991. We have also done a validity study to compare the electronic data of measurements of height and weight with the same visit's measurements of height and weight by trained health workers among 200 pregnant women and 160 children aged ≤2 years in four different local health centers. The correlations between electronic data and measurement data for body weight are 0.998 for pregnant women and 0.999 for children and for height/recumbent length are 0.997 for pregnant women and 0.999 for children.

Body mass index (BMI) was calculated by dividing weight in kilograms by the square of height in meters. Prepregnancy BMI was classified as normal weight (BMI < 24 kg/m^2^), overweight (BMI 24–27.9 kg/m^2^), and obese (BMI ≥ 28 kg/m^2^) using the Chinese BMI classification standard [17]. Weight gain of mothers during pregnancy was calculated as the difference between prepregnancy and delivery weight. We categorized women as having gained inadequate, adequate, or excessive weight according to 2009 American Institute of Medicine guidelines for weight gain during pregnancy [18]. Z-scores for birth weight-for-gestational age, birth length-for-gestational age, and birth weight-for-length were calculated using our own study population mean and standard deviations. Z-scores for weight-for-age, height-for-age, and weight-for-length were calculated based on the standards for the WHO growth reference [19].

### 2.4. Statistical Analyses

The general characteristics of both mothers and children according to different maternal glucose concentrations at 26–30 gestational weeks were compared using General Linear Model and chi-square test. General Linear Models were used to compare the differences in (1) Z-scores for birth length and birth weight-for-length; (2) Z-scores for length-for-age and weight-for-length at months 3, 6, 9, and 12; and (3) changes in Z-scores for length-for-age and weight-for-length for each three months and from birth to month 12, according to different maternal glucose concentrations at 26–30 gestational weeks. We included 3 multivariable models in the analyses. Model 1 was adjusted for maternal characters including maternal age, prepregnancy BMI, weight gain during pregnancy, family history of diabetes, education, and family income. To explore the potential mediating effect, in model 2 we additionally adjusted for infant feeding status; and in model 3 we additionally adjusted for birth variables for gestational age Z-score. All statistical analyses were performed with PASW for Windows, version 20.0 (Statistics 20, SPSS, IBM, USA).

## 3. Results

The general characteristics of both mothers and children according to different maternal glucose concentrations at 26–30 gestational weeks are presented in Table 1. Compared with mothers with normal glucose tolerance, mothers with GDM were older, had a higher prepregnancy BMI, and had more inadequate weight gain during pregnancy.

**Figure 1:**
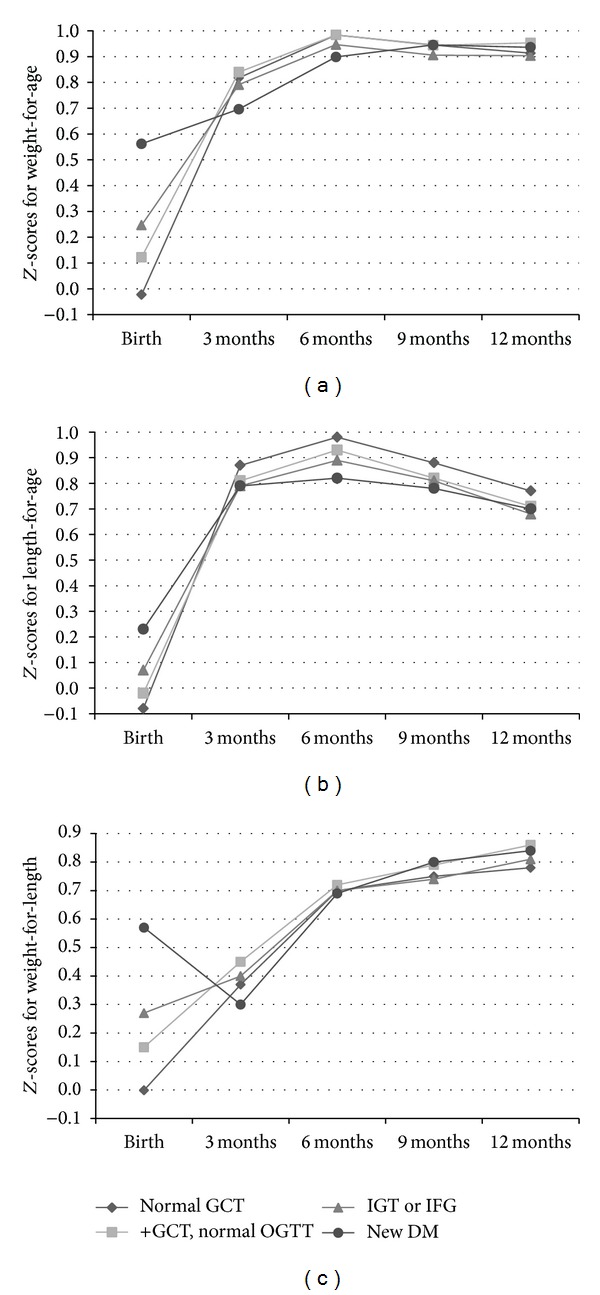
Comparison of Z-scores for body weight (a), body length (b), and weight-for-length (c) from birth to months 3, 6, 9, and 12 according to maternal OGTT at 26–30 gestational weeks.

Maternal glucose concentrations at 26–30 gestational weeks were positively associated with Z-scores for birth length-for-gestational age and birth weight-for-length (Table 2). Compared with infants born to mothers with normal glucose tolerance, infants born to mothers with GDM had higher mean values of Z-scores for birth length (new DM 0.23, IGT/IFG 0.07, and normal group −0.08, *P* < 0.001) and birth weight-for-length (new DM 0.57, IGT/IFG 0.27, normal group −0.001, *P* < 0.001), and lower mean values of Z-scores for length-for-age at months 3 (new DM 0.79, IGT/IFG 0.79, normal group 0.87, *P* = 0.007), 6 (new DM 0.82, IGT/IFG 0.89, normal group 0.98, *P* = 0.006), 9 (new DM 0.78, IGT/IFG 0.81, normal group 0.88, *P* = 0.007), and 12 (new DM 0.70, IGT/IFG 0.68, normal group 0.77, *P* = 0.002) (Table 2), especially among girls (online Table 1 see Supplementary Materials available online at: http://dx.doi.org/10.1155/2014/516980). We also compared mean values of Z-scores for weight-for-age from birth to months 3, 6, 9, and 12, and the results were similar to Z-scores for length-for-age and weight-for-length (Figure 1).

Table 3 presents the changes in Z-scores for length-for-age and weight-for-length for each three months and from birth to month 12 according to maternal OGTT glucose concentrations at 26–30 gestational weeks, and these results analyzed by gender are presented in online Table 2. After adjustment for maternal age, prepregnancy BMI, weight gain during pregnancy, family history of diabetes, education, family income (multivariable model 1), and infant feeding status (model 2), infants born to mothers with GDM had smaller changes in mean values of Z-scores for length-for-age and weight-for length from birth to month 3 and from birth to month 12 (all *P* < 0.001) compared with those infants born to mothers with normal glucose tolerance. From month 9 to 12, changes in mean values of Z-scores for weight-for-length were bigger among infants born to mothers with GDM (new DM 0.12, IGT/IFG 0.07) compared with those infants born to mothers with normal glucose tolerance (0.02). After additional adjustment for birth variables for gestational age Z-score (model 3), the smaller changes in mean values were still significant for length-for-age Z-score (*P* < 0.001) but were not significant for weight-for-length Z-score (*P* = 0.056) from birth to month 12 among infants born to mothers with new DM compared with those infants born to mothers with normal glucose tolerance. We did not find significant differences in changes in Z-scores for body length and weight-for-length from months 3 to 9 according to maternal OGTT glucose concentrations at 26–30 gestational weeks. Considering the effect of previous weight or length, we analyzed percentage change of Z-scores (change in Z-score between present and previous Z-scores accounting for previous Z-score) for weight-for-age and length-for-age for each three months from birth to month 12 according to maternal OGTT glucose concentrations at 26–30 gestational weeks (online Figure 1). The percentage change in Z-scores for weight-for-age from month 3 to 6 and from month 6 to 9 and the percentage change in Z-scores for length-for-age after month 6 were bigger among infants born to mothers with new DM compared with those infants born to mothers with normal glucose tolerance.

## 4. Discussion

The present study indicated that abnormal maternal glucose tolerance at 26–30 gestational weeks was associated with larger birth weight and birth length, less weight and length gain in the first 3 months of life, and more weight gain in the months 9–12 of life.

Previous studies have suggested that exposure to the intrauterine diabetic environment causes larger offspring size and more fatness at birth and higher risk of childhood obesity and young adult IGT and diabetes in the offspring [8, 20]. The offspring of Pima Indian women with preexistent type 2 diabetes and GDM were heavier at birth and had much higher rates of obesity at age 5–19 than the offspring of women with prediabetes or without diabetes [21]. However, at what age this association becomes apparent is unknown because most of these studies include the GDM's offspring older than 5 years old [8, 22]. Thus, there is a need to evaluate the effects of exposure to diabetes in utero on offspring's health status among ethnically diverse children with different age ranges, especially less than 5 years old [8]. The present study indicated that abnormal maternal glucose tolerance during pregnancy was associated with larger birth weight and birth length, lower length-for-age Z-score at months 3, 6, 9, and 12 of age, and more weight gain in the months 9–12 of life.

The present study is, to our knowledge, the first large prospective study to assess the associations of maternal glucose tolerance during pregnancy with changes in Z-scores for length-for-age and weight-for-length each three months from birth to month 12 in China. We firstly explored the potential mediating effects of fetal growth on the associations between maternal OGTT glucose concentrations and changes in Z-scores from birth to month 3 and found that abnormal maternal glucose tolerance during pregnancy was associated with slower weight gain and length gain in the first 3 months of life, which was consistent with earlier findings from the Project Viva that GDM predicted a slower gain in weight-for-length Z-scores in the first 6 months of life [12]. The Project Viva also found that higher cord blood leptin levels were associated with larger size at birth but less weight gain in the first 6 months of life [23]. In addition, we evaluated the effects of maternal OGTT glucose concentrations on changes in Z-scores each three months from month 3 to 12, which could partly decrease the potential mediating effects of fetal growth on above associations. Although we did not find any significant differences in weight-for-length Z-scores from month 3 to 9 among infants born to mothers with GDM, these infants had more gain in weight-for-length Z-scores from month 9 to 12 compared with infants born to mothers with normal glucose tolerance (Table 3). Since our birth cohort is still ongoing, we can assess the association of maternal OGTT glucose levels at 26–30 gestational weeks with offspring growth and development until 7 years old in the near future. The results of the present study promise to unravel some knotty questions in the early origins of obesity and to open up new potential avenues for primordial obesity prevention.

It is noteworthy that our study found the trend of rapid gain in weight-for-length Z-scores only among infants born to mothers with GDM after 9 months of birth. This phenomenon may relate with complementary food supplements for infants more than 6–9 months old. In animal models, maternal hyperglycemia resulted in perinatal hyperglycemia and increased hypothalamic insulin levels, followed by findings of permanent dysplasia of hypothalamic nuclei regulating food intake and metabolism in the offspring [24]. These alterations may increase food intake, with preference for fat, and further increase the risk of overweight or obesity in offspring at adulthood [24, 25]. Thus, early infant diet on the growth and development of children who may be programmed for a faster growth trajectory may be decided by utero exposure to overnutrition from a diabetic pregnancy.

We found that there was less breastfeeding among infants born to mothers with GDM than infants born to mothers with normal glucose during pregnancy, and offspring of mothers with GDM had more weight gain in the months 9–12 of life. One study with data from a retrospective cohort study conducted in Colorado (Exploring Perinatal Outcomes among Children, EPOCH) reported that the BMI trajectory was slower among infants in the adequate breastfeeding category (≥6 breast milk-months) than those infants in the low breastfeeding category (<6 breast milk-months) between birth and 9 months of age and between 4 and 6 years of age, and in both the offspring of diabetic pregnancies and offspring of nondiabetic pregnancies [26]. Many studies have shown that formula feeding rather than breastfeeding was associated with a rapid weight gain in early infancy and a higher risk of later obesity. The macronutrient compositions of breast milk (i.e., proteins, fat, and carbohydrate), not present in formula, may have a protective effect on metabolic programming and regulation of body fatness and growth rates [27]. Other studies suggested that breastfeeding might actually accelerate weight and length gain in the first few months [28, 29]. In our study, infant feeding status was considered as a potential medial factor in the association of maternal OGTT glucose concentrations with children growth and development in the multivariable analyses but the association of maternal OGTT glucose with children growth and development did not significant change after adjustment for infant feeding status. The present study is an ongoing project, and long follow-up of our study will assess association between breastfeeding and long-term effects of childhood BMI growth that extend beyond infancy into early and late childhood. Similar to Project Viva, we also found that the effect estimates for GDM were modestly attenuated after adjustment for birth variables for gestational age Z-score [12]. However, it is not clear for the effect of birth size on later growth.

There are several strengths in our study, including the large sample size of more than 27,000 mother-infant pairs in which OGTT was performed, repeated direct measures of maternal weight during pregnancy, repeated direct measures of the growth and development of infants at birth and each 3 months until 1 year old, and a wide range of potential confounders. Our main outcomes included both infant growth from birth to months 3, 6, 9, and 12 and infant growth each 3 months from month 3 to 12. A limitation of our study is that we only followed infant growth to 12 months old. Thus we cannot assess the effect of maternal OGTT glucose concentrations on offspring's growth and development after 12 months. However, we will get the children's later growth and development information in the near future.

In summary, our study indicated that abnormal maternal glucose tolerance during pregnancy was associated with higher birth weight and birth length, less weight gain and length gain in the first 3 months of life, and more weight gain in the months 6–12 of life. Intensified intervention of GDM during pregnancy should be taken to reduce abnormal glucose metabolism to decrease the risk of infants who are born to women with GDM becoming overweight or obese in later life.

## Supplementary Material

Compared with boys and girls born to mothers with normal glucose tolerance, boys and girls born to mothers with GDM had higher mean values of Z-scores for birth length and birth weight-for-length (online Table 1).

## Figures and Tables

**Table 1:** Characteristics of 27,157 mother-infant pairs according to maternal oral glucose tolerance test (OGTT) at 26–30 gestational weeks in Tianjin, China.

	Total	Maternal OGTT at 26–30 gestational weeks				*P* for differences
		Normal GCT	+GCT and normal OGTT	IGT or IFG	New DM	
Number of subjects	27 157	23 508	2229	1276	144	
Maternal characteristics						
Maternal age before pregnancy, y	28.0 (2.9)	27.9 (2.9)	28.2 (3.0)	29.0 (3.1)	29.4 (3.5)	<0.001
Gestational age at delivery, wk	39.1 (1.4)	39.1 (1.4)	39.0 (1.4)	38.9 (1.5)	38.3 (1.7)	<0.001
Systolic blood pressure during third trimester, mmHg	108 (10.7)	108 (10.6)	109 (10.9)	110 (11.5)	113 (10.5)	<0.001
Prepregnancy BMI, kg/m^2^	22.1 (3.4)	22.0 (3.3)	22.9 (3.6)	23.4 (3.5)	26.0 (4.0)	<0.001
Prepregnancy BMI category, *n* (%)						<0.001
Underweight	2976 (11.0)	2742 (11.7)	171 (7.7)	62 (4.9)	1 (0.7)	
Normal weight	17 518 (64.5)	15 432 (65.6)	1322 (59.2)	709 (55.5)	55 (38.2)	
Overweight	5059 (18.6)	4096 (17.4)	543 (24.4)	375 (29.4)	45 (31.2)	
Obesity	1604 (5.9)	1238 (5.3)	193 (8.7)	130 (10.2)	43 (29.9)	
Gestational weight gain (IOM category), *n* (%)						<0.001
Excessive	21 466 (79.0)	18 689 (79.5)	1739 (78.0)	928 (72.7)	110 (76.4)	
Adequate	4287 (15.8)	3654 (15.5)	366 (16.4)	247 (19.4)	20 (13.9)	
Inadequate	1404 (5.2)	1165 (5.0)	124 (5.6)	101 (7.9)	14 (9.7)	
Mother's education, *n* (%)						0.152
University and above	13 624 (50.1)	11 811 (50.2)	1087 (48.8)	664 (52.1)	62 (43.0)	
Junior college	7596 (28.0)	6577 (28.0)	629 (28.2)	350 (27.4)	40 (27.8)	
High school and under	5937 (21.9)	5120 (21.8)	513 (23.0)	262 (20.5)	42 (29.2)	
Family income, yuan/month*, *n* (%)						0.073
≥3000	15 878 (58.5)	13 739 (58.4)	1282 (57.5)	784 (61.5)	73 (50.7)	
2000–2999	6087 (22.4)	5265 (22.4)	501 (22.5)	281 (22.0)	40 (27.8)	
≤1999	5192 (19.1)	4504 (19.2)	446 (20.0)	211 (16.5)	31 (21.5)	
Child characteristics						
Boy, *n* (%)	14 090 (51.9)	12 147 (51.7)	1178 (52.8)	684 (53.6)	81 (56.2)	0.276
Mode of infant feeding, *n* (%)						0.048
Exclusive breastfeeding	4824 (17.8)	4201 (17.9)	397 (17.8)	209 (16.4)	17 (11.8)	
Mixed breast and formula	18 940 (69.7)	16 400 (69.7)	1560 (70.0)	880 (69.0)	100 (69.4)	
Weaned from breastfeeding	2887 (10.6)	2485 (10.6)	224 (10.0)	155 (12.1)	23 (16.0)	
Exclusive formula feeding	506 (1.9)	422 (1.8)	48 (2.2)	32 (2.5)	4 (2.8)	
Weight, kg						
Birth	3.39 (0.47)	3.38 (0.46)	3.42 (0.48)	3.45 (0.51)	3.50 (0.63)	<0.001
3 months	6.93 (0.81)	6.93 (0.81)	6.95 (0.80)	6.92 (0.81)	6.83 (0.80)	0.343
6 months	8.67 (1.02)	8.67 (1.02)	8.68 (1.00)	8.64 (1.02)	8.63 (1.04)	0.711
9 months	9.69 (1.11)	9.69 (1.11)	9.70 (1.10)	9.66 (1.11)	9.73 (1.16)	0.717
12 months	10.5 (1.16)	10.5 (1.16)	10.5 (1.13)	10.5 (1.14)	10.5 (1.32)	0.272
Length, cm						
Birth	50.1 (1.6)	50.1 (1.6)	50.1 (1.7)	50.2 (1.6)	50.2 (2.1)	0.046
3 months	62.9 (2.3)	63.0 (2.3)	62.8 (2.3)	62.8 (2.3)	62.8 (2.7)	0.042
6 months	69.2 (2.5)	69.2 (2.5)	69.1 (2.4)	69.0 (2.5)	69.0 (2.5)	0.070
9 months	73.5 (2.6)	73.5 (2.6)	73.4 (2.5)	73.4 (2.6)	73.3 (2.5)	0.058
12 months	77.1 (2.7)	77.1 (2.7)	76.9 (2.6)	76.9 (2.6)	77.0 (2.8)	0.017

Data are mean (SD) or percent.

GCT: glucose challenge test; IFG: impaired fasting glucose; IGT: impaired glucose tolerance; New DM: newly diagnosed diabetes; BMI: body mass index.

Normal GCT was defined as a glucose concentration <7.8 mmol/L after the GCT; +GCT and normal OGTT were defined as failed GCT (a glucose concentration ≥7.8 mmol/L after the GCT) with normal glucose after a 75 g 2-hour OGTT (fasting glucose <6.1 mmol/L and 2-hour glucose <7.8 mmol/L); IGT was defined as failed GCT and fasting glucose <7.0 mmol/L and 2-hour glucose ≥7.8 and <11.1 mmol/L; IFG was defined as failed GCT and fasting glucose ≥6.1 and <7.0 mmol/L and 2-hour glucose <7.8 mmol/L; and new DM was defined as failed GCT and fasting glucose ≥7.0 mmol/L or 2-hour glucose ≥11.1 mmol/L.

**Table 2:** Comparison of Z-scores for body length and weight-for-length from birth to months 3, 6, 9, and 12 according to maternal OGTT at 26–30 gestational weeks.

	Total	Maternal OGTT at 26–30 gestational weeks				*P* for differences
		Normal GCT	+GCT and normal OGTT	IGT or IFG	New DM	
Birth for gestational weeks						
Number of subjects	27 157	23 508	2229	1276	144	
Length-for-gestational age Z-score	−0.06 (0.91)	−0.08 (0.90)	−0.02 (0.91)	0.07 (0.91)	0.23 (1.16)	<0.001
Weight-for-length Z-score	0.03 (1.00)	−0.001 (0.98)	0.15 (1.01)	0.27 (1.08)	0.57 (1.27)	<0.001
3 months						
Number of subjects	24 722	21 421	2017	1154	130	
Length-for-age Z-score	0.86 (1.03)	0.87 (1.03)	0.81 (1.02)	0.79 (1.02)	0.79 (1.20)	0.007
Weight-for-length Z-score	0.38 (1.04)	0.37 (1.04)	0.45 (1.06)	0.40 (1.06)	0.30 (1.07)	0.007
6 months						
Number of subjects	25 400	21 960	2110	1195	135	
Length-for-age Z-score	0.97 (1.06)	0.98 (1.05)	0.93 (1.05)	0.89 (1.05)	0.82 (1.07)	0.006
Weight-for-length Z-score	0.70 (1.04)	0.70 (1.03)	0.72 (1.08)	0.70 (1.07)	0.69 (1.13)	0.701
9 months						
Number of subjects	22 920	19 820	1897	1089	114	
Length-for-age Z-score	0.87 (1.04)	0.88 (1.04)	0.82 (1.03)	0.81 (1.04)	0.78 (1.00)	0.007
Weight-for-length Z-score	0.76 (1.00)	0.75 (1.00)	0.79 (1.00)	0.74 (1.02)	0.80 (1.05)	0.425
12 months						
Number of subjects	23 444	20 203	1988	1130	123	
Length-for-age Z-score	0.76 (1.04)	0.77 (1.04)	0.71 (1.01)	0.68 (1.00)	0.70 (1.08)	0.002
Weight-for-length Z-score	0.79 (0.98)	0.78 (0.98)	0.86 (0.96)	0.81 (0.98)	0.84 (1.09)	0.002

Data are mean (SD).

OGTT: oral glucose tolerance test; IFG: impaired fasting glucose; IGT: impaired glucose tolerance; New DM: newly diagnosed diabetes mellitus.

**Table 3:** Changes in Z-scores for body length-for-age and weight-for-length for each three months and from birth to month 12 according to maternal OGTT at 26–30 gestational weeks.

Changes in Z-scores	Maternal OGTT at 26–30 gestational weeks				*P* for differences
	Normal GCT	+GCT and normal OGTT	IGT or IFG	New DM	
From 0 to 3 months					
No. of subjects	21 419	2017	1154	130	
Length-for-age					
Model 1*	0.94 (0.01)	0.86 (0.03)	0.75 (0.03)	0.62 (0.10)	<0.001
Model 2^†^	0.94 (0.01)	0.85 (0.03)	0.76 (0.03)	0.63 (0.10)	<0.001
Model 3^§^	0.94 (0.01)	0.87 (0.02)	0.82 (0.03)	0.76 (0.09)	<0.001
Weight-for-length					
Model 1*	0.37 (0.01)	0.32 (0.03)	0.18 (0.04)	−0.17 (0.11)	<0.001
Model 2^†^	0.37 (0.01)	0.32 (0.03)	0.18 (0.04)	−0.16 (0.11)	<0.001
Model 3^§^	0.35 (0.01)	0.39 (0.02)	0.33 (0.03)	0.14 (0.09)	0.027
From 3 to 6 months					
No. of subjects	20 366	1932	1102	124	
Length-for-age					
Model 1*	0.11 (0.01)	0.11 (0.02)	0.09 (0.03)	0.02 (0.08)	0.584
Model 2^†^	0.11 (0.01)	0.12 (0.02)	0.09 (0.03)	0.02 (0.08)	0.519
Model 3^§^	0.11 (0.01)	0.12 (0.02)	0.09 (0.03)	0.02 (0.08)	0.609
Weight-for-length					
Model 1*	0.32 (0.01)	0.28 (0.02)	0.29 (0.03)	0.38 (0.08)	0.107
Model 2^†^	0.32 (0.01)	0.28 (0.02)	0.29 (0.03)	0.38 (0.08)	0.108
Model 3^§^	0.32 (0.01)	0.28 (0.02)	0.28 (0.03)	0.37 (0.08)	0.075
From 6 to 9 months					
No. of subjects	18 992	1827	1035	110	
Length-for-age					
Model 1*	−0.09 (0.01)	−0.11 (0.02)	−0.09 (0.02)	−0.07 (0.07)	0.838
Model 2^†^	−0.09 (0.01)	−0.11 (0.02)	−0.10 (0.02)	−0.07 (0.07)	0.839
Model 3^§^	−0.09 (0.01)	−0.10 (0.02)	−0.09 (0.02)	−0.07 (0.07)	0.860
Weight-for-length					
Model 1*	0.05 (0.01)	0.06 (0.02)	0.03 (0.02)	0.13 (0.07)	0.576
Model 2^†^	0.05 (0.01)	0.06 (0.02)	0.03 (0.02)	0.12 (0.07)	0.580
Model 3^§^	0.05 (0.01)	0.06 (0.02)	0.03 (0.02)	0.12 (0.07)	0.562
From 9 to 12 months					
No. of subjects	17 778	1739	1013	99	
Length-for-age					
Model 1*	−0.12 (0.01)	−0.14 (0.02)	−0.14 (0.02)	−0.10 (0.07)	0.662
Model 2^†^	−0.12 (0.01)	−0.14 (0.02)	−0.14 (0.02)	−0.10 (0.07)	0.663
Model 3^§^	−0.12 (0.01)	−0.14 (0.02)	−0.14 (0.02)	−0.10 (0.07)	0.688
Weight-for-length					
Model 1*	0.02 (0.01)	0.07 (0.02)	0.07 (0.02)	0.12 (0.08)	0.011
Model 2^†^	0.02 (0.01)	0.07 (0.02)	0.07 (0.02)	0.12 (0.08)	0.012
Model 3^§^	0.02 (0.01)	0.07 (0.02)	0.07 (0.02)	0.12 (0.08)	0.013
From 0 to 12 months					
No. of subjects	20 201	1988	1130	123	
Length-for-age					
Model 1*	0.85 (0.01)	0.73 (0.03)	0.63 (0.04)	0.51 (0.11)	<0.001
Model 2^†^	0.85 (0.01)	0.73 (0.03)	0.63 (0.04)	0.50 (0.11)	<0.001
Model 3^§^	0.84 (0.01)	0.75 (0.02)	0.69 (0.03)	0.62 (0.09)	<0.001
Weight-for-length					
Model 1*	0.77 (0.01)	0.71 (0.03)	0.56 (0.04)	0.29 (0.11)	<0.001
Model 2^†^	0.77 (0.01)	0.71 (0.03)	0.56 (0.04)	0.29 (0.11)	<0.001
Model 3^§^	0.76 (0.01)	0.79 (0.02)	0.71 (0.03)	0.61 (0.09)	0.056

Data are mean (SE).

IFG: impaired fasting glucose; IGT: impaired glucose tolerance; New DM: newly diagnosed diabetes mellitus.

*Model 1 was adjusted for maternal age, prepregnancy BMI, weight gain during pregnancy, family history of diabetes, education of mother, and income.

^†^Model 2 was adjusted for above variables and also mode of infant feeding.

^§^Model 3 was adjusted for above variables and also birth length-for-gestational age Z-score in change in length-for-age Z-score, birth weight-for-birth length Z-score in change in weight-for-length Z-score.
