# Supplementary material for: Maternal OGTT Glucose Levels at 26–30 Gestational Weeks with Offspring Growth and Development in Early Infancy
Source: Biomed Res Int. 2014 Feb 13;2014:516980. doi: 10.1155/2014/516980 (PMC3943263; doi:10.1155/2014/516980)
Supplement: Supplementary file 1 — Compared with boys and girls born to mothers with normal glucose tolerance, boys and girls born to mothers with GDM had higher mean values of Z-scores for birth length and birth weight-for-length (online Table 1). [file 516980.f1.pdf]

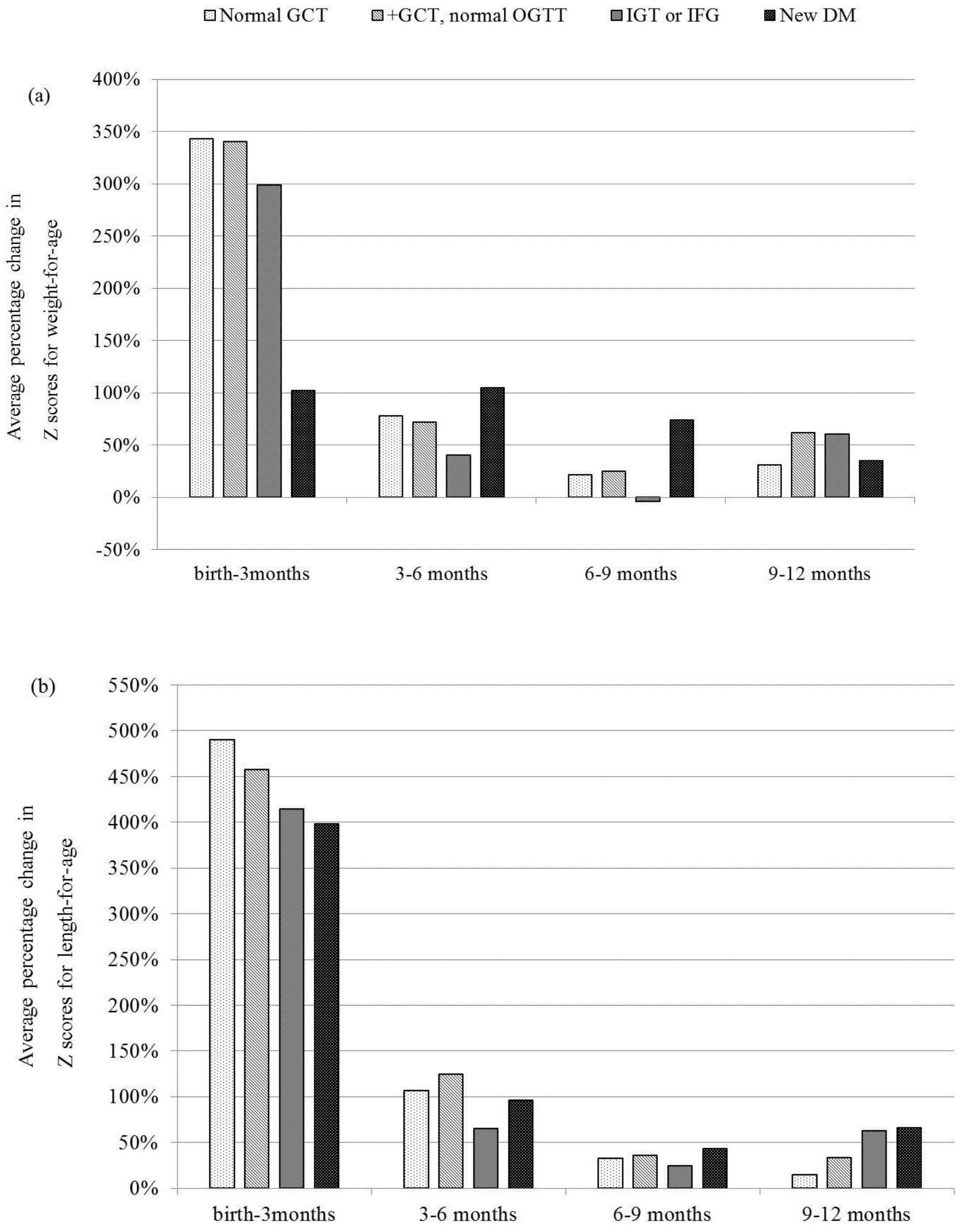

Online Figure 1: Average percentage change in Z-scores for body weight (a) and body length (b) from birth to months 3, 6, 9, and 12 according to maternal OGTT at 26-30 gestational weeks

Online Table 1. Comparison of Z-scores for body length and weight-for-length by gender from birth to months 3, 6, 9, and 12 according to maternal OGTT at 26-30 gestational weeks

	Total	Maternal OGTT at 26-30 gestational weeks				P for differences
		Normal GCT	+GCT, normal OGTT	IGT or IFG	New DM	
Birth for gestational weeks						
Boys						
No. of subjects	14 090	12 147	1 178	684	81	
Length-for-gestational age z-score	-0.06 (0.90)	-0.08 (0.90)	-0.03 (0.86)	0.06 (0.91)	0.25 (1.21)	<0.001
Weight-for-length z-score	0.02 (1.00)	-0.01 (0.99)	0.12 (0.98)	0.26 (1.08)	0.52 (1.36)	<0.001
Girls						
No. of subjects	13 067	11 361	1 051	592	63	
Length-for-gestational age z-score	-0.07 (0.91)	-0.08 (0.91)	-0.01 (0.96)	0.07 (0.91)	0.20 (1.10)	<0.001
Weight-for-length z-score	0.03 (1.00)	0.004 (0.98)	0.19 (1.04)	0.27 (1.08)	0.64 (1.15)	<0.001
3 month						
Boys						

No. of subjects	12 803	11 042	1 073	619	69	
Length-for-gestational age z-score	0.80 (1.06)	0.81 (1.06)	0.75 (1.02)	0.77 (1.08)	0.87 (1.26)	0.261
Weight-for-length z-score	0.44 (1.08)	0.44 (1.07)	0.51 (1.08)	0.45 (1.14)	0.20 (1.04)	0.053
Girls						
No. of subjects	11 919	10 379	944	535	61	
Length-for-gestational age z-score	0.93 (0.99)	0.94 (0.99)	0.89 (1.02)	0.81 (0.93)	0.71 (1.12)	0.005
Weight-for-length z-score	0.32 (1.00)	0.31 (1.00)	0.39 (1.02)	0.35 (0.97)	0.40 (1.11)	0.063
6 month						
Boys						
No. of subjects	13 137	11 300	1 121	641	75	
Length-for-gestational age z-score	0.92 (1.07)	0.93 (1.08)	0.85 (1.02)	0.89 (1.11)	0.81 (1.14)	0.070
Weight-for-length z-score	0.75 (1.07)	0.75 (1.07)	0.77 (1.11)	0.72 (1.14)	0.63 (1.18)	0.667
Girls						
No. of subjects	12 263	10 660	989	554	60	
Length-for-gestational age z-score	1.02 (1.03)	1.03 (1.02)	1.03 (1.08)	0.90 (0.99)	0.83 (0.98)	0.014

Weight-for-length z-score	0.65 (0.99)	0.64 (0.98)	0.67 (1.04)	0.67 (1.00)	0.77 (1.07)	0.539
9 month						
Boys						
No. of subjects	11 892	10 221	1 017	588	66	
Length-for-gestational age z-score	0.82 (1.08)	0.83 (1.08)	0.76 (1.06)	0.79 (1.11)	0.81 (1.08)	0.302
Weight-for-length z-score	0.80 (1.04)	0.79 (1.03)	0.82 (1.04)	0.79 (1.10)	0.85 (1.07)	0.803
Girls						
No. of subjects	11 028	9 599	880	501	48	
Length-for-gestational age z-score	0.93 (1.00)	0.94 (1.00)	0.88 (1.00)	0.83 (0.96)	0.74 (0.90)	0.016
Weight-for-length z-score	0.71 (0.95)	0.71 (0.95)	0.75 (0.95)	0.69 (0.91)	0.73 (1.04)	0.615
12 month						
Boys						
No. of subjects	12 165	10 437	1 054	607	67	
Length-for-gestational age z-score	0.72 (1.08)	0.72 (1.08)	0.67 (1.03)	0.65 (1.10)	0.68 (1.16)	0.186
Weight-for-length z-score	0.82 (1.02)	0.81 (1.02)	0.87 (0.99)	0.85 (1.05)	0.88 (1.16)	0.240

Girls						
No. of subjects	11 279	9 766	934	523	56	
Length-for-gestational age z-score	0.81 (1.00)	0.82 (1.00)	0.75 (1.00)	0.70 (0.88)	0.72 (0.99)	0.015
Weight-for-length z-score	0.75 (0.94)	0.74 (0.94)	0.85 (0.93)	0.77 (0.89)	0.80 (1.00)	0.006

Data are means (SD).

OGTT, oral glucose tolerance test; IFG, impaired fasting glucose; IGT, impaired glucose tolerance; New DM, newly diagnosed diabetes mellitus.

Online Table 2. Changes in Z-scores for body length for age and weight for length by gender for each three months and from birth to months 12 according to maternal OGTT at 26-30 gestational weeks

	Maternal OGTT at 26-30 gestational weeks				P for
Changes in Z-scores	Normal GCT	+GCT, normal OGTT	IGT or IFG	New DM	differences
From 0 to 3 months					
Boys					
No. of subjects	11 041	1 073	619	69	
Length-for-age	0.88 (0.01)	0.80 (0.03)	0.80 (0.04)	0.81 (0.12)	0.037
Weight-for-length	0.43 (0.01)	0.41 (0.04)	0.23 (0.05)	-0.20 (0.16)	<0.001
Girls					
No. of subjects	10 378	944	535	61	
Length-for-age	1.01 (0.01)	0.94 (0.03)	0.83 (0.04)	0.69 (0.12)	<0.001
Weight-for-length	0.30 (0.01)	0.21 (0.04)	0.13 (0.06)	-0.12 (0.16)	<0.001
From 3 to 6 months					
Boys					
No. of subjects	10 464	1 031	591	65	
Length-for-age	0.12 (0.01)	0.09 (0.03)	0.11 (0.04)	-0.09 (0.11)	0.179
Weight-for-length	0.31 (0.01)	0.26 (0.03)	0.27 (0.04)	0.45 (0.12)	0.169
Girls					
No. of subjects	9 902	901	511	59	
Length-for-age	0.10 (0.01)	0.14 (0.03)	0.06 (0.04)	0.13 (0.11)	0.315
Weight-for-length	0.33 (0.01)	0.30 (0.03)	0.31 (0.04)	0.29 (0.11)	0.583
From 6 to 9 months					

Boys					
No. of subjects	9 764	983	562	63	
Length-for-age	-0.09 (0.01)	-0.09 (0.03)	-0.11 (0.03)	-0.13 (0.10)	0.916
Weight-for-length	0.05 (0.01)	0.06 (0.03)	0.05 (0.03)	0.22 (0.10)	0.367
Girls					
No. of subjects	9 228	844	473	47	
Length-for-age	-0.08 (0.01)	-0.13 (0.03)	-0.07 (0.03)	0.001 (0.11)	0.334
Weight-for-length	0.06 (0.01)	0.06 (0.03)	0.004 (0.03)	-0.01 (0.11)	0.387
From 9 to 12 months					
Boys					
No. of subjects	9 172	926	548	56	
Length-for-age	-0.11 (0.01)	-0.11 (0.03)	-0.14 (0.03)	-0.09 (0.10)	0.841
Weight-for-length	0.02 (0.01)	0.05 (0.03)	0.06 (0.03)	0.14 (0.10)	0.333
Girls					
No. of subjects	8 606	813	465	43	
Length-for-age	-0.13 (0.01)	-0.16 (0.03)	-0.13 (0.03)	-0.12 (0.11)	0.558
Weight-for-length	0.03 (0.01)	0.10 (0.03)	0.07 (0.03)	0.09 (0.11)	0.026
From 0 to 12 months					
Boys					
No. of subjects	10 436	1 054	607	67	
Length-for-age	0.80 (0.01)	0.70 (0.04)	0.62 (0.05)	0.50 (0.15)	<0.001
Weight-for-length	0.82 (0.01)	0.76 (0.04)	0.59 (0.05)	0.36 (0.15)	<0.001
Girls					

No. of subjects	9 765	934	523	56	
Length-for-age	0.89 (0.01)	0.77 (0.04)	0.64 (0.05)	0.51 (0.16)	<0.001
Weight-for-length	0.72 (0.01)	0.66 (0.04)	0.51 (0.05)	0.20 (0.16)	<0.001

Data are means (SE). IFG, impaired fasting glucose; IGT, impaired glucose tolerance; New DM, newly diagnosed diabetes mellitus.

Model was adjusted for maternal age, prepregnancy BMI, weight gain during pregnancy, family history of diabetes, education of mother, income, mode of infant feeding, and birth length-for-gestational age Z-score in change length-for-age Z-score, birth weight-for-birth length Z-score in change weight-for-length Z-score.
